# Modelling child anaemia and co-existing infections using log-linear models

**DOI:** 10.1186/s12936-025-05281-1

**Published:** 2025-03-07

**Authors:** Grace Kakaire, Gregory Kerich, Robert Too, Mathew Kosgei

**Affiliations:** 1 School of Statistics and Planning, Makerere University, Kampala, Uganda; 2 School of Science and Aerospace Studies, Moi University, Eldoret, Kenya

**Keywords:** Association, Homogenous, Independent, Saturated, Uganda

## Abstract

**Background:**

Uganda grapples with a considerable anaemia-malaria-fever burden, reporting approximate prevalence rates as high as 33%, 34%, and 37% in specific regions. In recent years, attempts have been made by the Ministry of Health to address the combined burden of the characterized conditions of these illnesses. However, the relationship between malaria, fever, and anaemia has not been well characterized among young children living in many communities. By employing log-linear models, this study aims to examine patterns and associations between malaria, fever, and child anaemia in Uganda while controlling for maternal anaemia.

**Methods:**

Utilizing secondary data from the 2018–2019 Uganda Malaria Indicator Survey (MIS), the study focused on children aged 0–60 months. The sample included 7,124 children selected through a two-stage sampling process involving clusters and households. Five log linear models, namely; saturated, mutual independence, joint independence, conditional independence and homogenous models were fitted. The saturated model was used as the reference model.

**Results:**

The *G*^2^ statistics and p-values for each model were as follows: saturated model (*G*^2^ = 0.00, p = 1.00), mutual independence model (*G*^2^ = 321.45, p < 0.001), joint independence model (*G*^2^ = 214, p < 0.001), conditional independence model (*G*^2^ = 109.53, p < 0.001), and homogeneous model (*G*^2^ = 10.24, p = 0.76). The homogeneous model adequately fit the data, showing the smallest *G*^2^ statistic and the largest p-value, indicating no significant lack of fit. Additionally, children who tested positive for malaria were found to be two times more likely to have anaemia than those who tested negative.

**Conclusion:**

This study underscores the interconnectedness of malaria, fever, and anaemia in Ugandan children, with maternal anaemia serving as a critical contextual factor. Using log-linear modelling, uncovered patterns and interactions that highlight how these conditions influence one another, emphasizing the value of integrated interventions. Targeted approaches that address maternal health, enhance malaria prevention, and provide nutritional support are essential to reducing the syndemic burden of these conditions in Uganda.

## Background

Childhood morbidity is a significant global health concern, especially in low- and middle-income countries where infectious diseases remain prevalent. Fever, malaria, and anaemia are among the leading causes of illness in children, contributing substantially to morbidity and child illnesses, often caused by bacterial, viral, or parasitic infections, are a primary cause of morbidity and mortality among children under five [1].

Anaemia, characterized by a reduction in the number of red blood cells or a decrease in haemoglobin concentration, is a common consequence of various health conditions. In children, anaemia is often linked to nutritional deficiencies, infectious diseases, and parasitic infections. It affects individuals of all ages, but children are disproportionately affected. Its impact extends beyond immediate health effects, affecting cognitive development and overall well-being of the child; making it a priority area for public health interventions especially in Sub-Saharan Africa [2, 3].

Anaemia in young children has previously been recommended as a key indicator to monitor the burden of malaria and fever; however, recent years have seen a decline in the awareness and reporting of this indicator [3, 4]. The surveillance of anaemia poses challenges due to its multiple causes in children [5]. In addition, the relationship between malaria and anaemia can be confounded by several factors, including nutritional deficiencies (specifically iron deficiency) and intestinal parasites, all of which contribute to anaemia in children [6]. Although the global burden of anaemia has improved significantly since 1990, anaemia in children has shown much less improvement, thus revealing inconsistencies in the efforts to prevent childhood anaemia [5]. This may also be attributed to the complex multifactorial causes of anaemia in children which require a solid understanding of their contribution to childhood anaemia.

More specifically, an understanding of the underlying co-infections and their relationship with anaemia in high-burden regions will aid in formulating a more targeted approach for anaemia control. Many studies have considered the determinants of anaemia, fever and malaria in children separately [2, 7, 8], and others have considered them as determinants of each other where children who tested positive for malaria were more than 3 times as likely to have anaemia. On the other hand, researchers have reported that those with anaemia were more than twice as likely to have malaria [9–13]. A cross-sectional study among Palestinian refugee children showed that a current episode of fever or diarrhoea was associated with an increased risk of anaemia [14].

Despite multi-sectoral efforts to reduce the burden of anaemia in Uganda, the 2016 Uganda Demographic and Health Survey (UDHS) reported that the prevalence of anaemia was 53% in children aged 0–59 months, up from 49% in 2011, and was 32% in women of reproductive age, up from 23% in 2011 [15]. Past studies in Uganda have examined anaemia, malaria, and fever using survey data for specific districts or regions, but independently [2, 16–18].

While each health condition has been studied individually, there is a growing recognition of their interconnectedness. Children in resource-limited settings [19] often face a complex syndemic nature of these health challenges in resource-limited settings, where co-infections and nutritional deficiencies often overlap [11]. By focusing on these interconnected conditions in young children, this research contributes to a more comprehensive understanding of their combined impact. This characterization is essential for informing targeted public health interventions and optimizing resource allocation in high-burden regions like Uganda. Understanding the complex relationships among fever, malaria, and anaemia is crucial for developing holistic and effective public health interventions. While the Ministry of Health has made significant efforts to address the combined burden of malaria, fever, and anaemia, the relationship between these conditions remains insufficiently characterized, particularly among young children in community settings. This study aims to address this gap by employing log-linear modelling to examine the intricate associations among malaria, fever, and child anaemia in Uganda. This approach makes it possible to explore not only the direct relationships between these conditions but also their potential interactions while accounting for maternal anaemia as an important contextual factor.

To unravel the intricate associations among these health conditions, log-linear modelling provides a powerful statistical approach [20]. This method allows for the exploration of dependencies and interactions between categorical variables, providing insights into the complex web of factors contributing to childhood morbidity. By employing log-linear models, the study, therefore, aims to examine patterns and associations between malaria, fever, and child anaemia in Uganda while controlling for maternal anaemia.

## Methods

### Data source and study population

Data used in this study were obtained from the 2018–19 Uganda Malaria Indicator Survey (MIS), which collected information on a nationally representative sample of women of childbearing age (15–49 years)**.** The MIS was based on a stratified two-stage cluster design [15]. In the first stage, 20 sampling strata were created, and 210 clusters were selected with probability-proportional-to-size sampling. At the second stage, using complete lists of households in the selected clusters, 28 households were chosen from each cluster with equal probability systematic sampling.

All women of age 15–49 years in the sampled households, who were either permanent residents or visitors in the household on the night preceding the survey, were eligible for interview. Similarly, all children of age less than 5 years were eligible for anaemia and malaria testing.

### Data collection and measurements

Blood samples were taken from the fingers or heels of children aged less than 5 years and tested on the spot using Rapid Diagnostic Tests (RDTs). In addition, thick and thin blood smears were prepared and tested by microscopy. Results were recorded as either positive or negative if malaria parasites were found or not in the blood sample, respectively. In addition, blood samples taken from finger or heel prick wounds were used to assess anaemia in all children between 6 and 59 months of age, with the consent of a parent or guardian in the study household; malaria was tested as well. This study is based on her 7,124 children who had valid responses to questions regarding the prevalence/status of (i) fever, (ii) anaemia, and (iii) malaria.

#### Methodological flowchart

Below is a flowchart summarizing the methodological approach, from data collection to model selection criteria:

**Study variables**

The study has three outcome variables of interest, namely.

(i) having had fever in the 2 weeks preceding the survey (F)
(ii) having had malaria in the 2 weeks preceding the survey (D)
(iii) maternal anaemia status (D)
(iv) child’s anaemia status (A)

The child’s and maternal anaemia status is based on the WHO definition of anaemia in children 0–59 months of age, with a haemoglobin concentration of less than 11 g/dL after adjustment for lying altitude, as measured by a portable HemoCue analyzer [21]. The child’s malaria status was based on Rapid Diagnostic Test (RDT) results. A drop of blood was tested for the presence of *Plasmodium* parasites using the SD Bioline Pf/Pv RDT. This type of test is increasingly used as a diagnostic test when reliable microscopy is not available [15].

### Statistical analysis

#### Addressing study objectives

This study employed log-linear modelling to explore the associations among child anaemia (A), malaria (D), and fever (F), while accounting for maternal anaemia (M). The primary objective was to evaluate patterns of association and determine the most parsimonious model. This is achieved using log-linear modelling to explore the main effects and interaction effects among these variables. Log-linear models analyse these associations by testing conditional and joint independencies, ensuring the results reflect the stated objectives. By comparing nested models and examining interaction terms, the analysis identifies the most parsimonious model that adequately represents the patterns and associations among these variables.

Maternal anaemia was treated as a control variable in the models by including it in all interaction terms and testing its conditional associations with child anaemia, malaria, and fever. Specifically, maternal anaemia was incorporated into the hierarchical log-linear models to assess its influence on the relationships among the other variables. This ensured that any observed patterns in child anaemia, malaria, and fever were evaluated while accounting for the potential confounding effects of maternal anaemia.

Each of the log-linear models contributes uniquely to analysing the main research question:

i. *Mutual Independence Model (A, D, M).* This simplest model assumes that child anaemia, malaria, and maternal anaemia are entirely independent of each other. It serves as a baseline for comparison to assess whether the variables exhibit any associations.
ii. *Joint Independence Model (e.g., AD, M).* This model assumes that two variables (e.g., child anaemia and malaria) are independent of the third (e.g., maternal anaemia). It helps identify cases where specific pairs of variables share an association while being independent of others.
iii. *Conditional Independence Model (e.g., AD, AM).* This model examines whether two variables are conditionally independent given the third. For instance, it can assess whether child anaemia and malaria are independent after accounting for maternal anaemia. This is crucial for understanding how maternal anaemia influences the relationships among the other variables.
iv. *Homogeneous Association Model (AD, AM, DM). This* model incorporates two-way interactions between all pairs of variables while assuming the conditional odds ratios are the same across levels of the third variable. It captures more complex relationships while remaining more parsimonious than the saturated model.
v. *Saturated Model (ADM).* This model includes all main effects, two-way interactions, and the three-way interaction. While it provides a perfect fit to the data, it may over fit and lack parsimony. It serves as the benchmark against which simpler models are evaluated.

##### Building log-linear models

Log-linear analysis examines associations between categorical variables by modelling main effects and interaction effects. All variables were treated as response variables. To examine the associations among malaria, fever, and child anaemia while controlling for maternal anaemia, each variable’s interaction and independence were assessed through the hierarchical log-linear models. The inclusion of maternal anaemia in the models allowed controlling for its influence on child anaemia and malaria.

The following log-linear models were analysed:

##### Saturated model

The saturated model includes all main effects, two way, and three-way interaction effects among the variables in the model. The saturated model serves as a baseline for comparison with more parsimonious models. It essentially fits the data perfectly, as it allows for all possible associations and interactions, but it may be too complex and overfit the data.

In this study, the saturated model with three-way effects of Child Anaemia status (A), Malaria status (D), and Maternal Anaemia status (M) shows the following model structure:

1 $$log\left( {\mu_{ijk} } \right) = \lambda + \lambda_{i}^{A} + \lambda_{j}^{D} + \lambda_{k}^{M} + \lambda_{ik}^{AM} + \lambda_{jk}^{DM} + \lambda_{ij}^{AD} + \lambda_{ijk}^{ADM}$$

where;

**Table:** 

$log\left({\mu }_{ijk}\right)$	$=$	log of the expected cell frequency of the cases for cell i, j and k in the contingency table
$\lambda$	$=$	overall effect or a grand mean of the logarithms of the expected frequency
${\lambda }_{i}^{A},{\lambda }_{j}^{D},{\lambda }_{k}^{M}$	$=$	the main effects of variables A, D and M
${\lambda }_{ik}^{AM},{\lambda }_{jk}^{DM},{\lambda }_{ij}^{AD}$	$=$	the interaction or association between two variables; A & M, D & M and A & D
${\lambda }_{ijk}^{ADM}$	$=$	the interaction or association between three variables A, D and M

#### Homogeneous association model

A homogeneous model exists when there is a two-way interaction. A homogeneous association occurs if the conditional odds ratios between any two of its variables are the same for every level of the third variable. The homogeneous model structure of Child Anaemia status (A), Malaria status (D) and Maternal Anaemia status (M) is as follows:

2 $$log\left( {\mu_{ijk} } \right) = \lambda + \lambda_{i}^{A} + \lambda_{j}^{D} + \lambda_{k}^{M} + \lambda_{ik}^{AM} + \lambda_{jk}^{DM} + \lambda_{ij}^{AD}$$

#### Independence models

##### Mutual independence model

This is the simplest model where all the variables are independent of one another. The model function (A, D, M) is:

3 $$log\left( {\mu_{ijk} } \right) = \lambda + \lambda_{i}^{A} + \lambda_{j}^{D} + \lambda_{k}^{M}$$

##### Joint independence model

This model indicates that two variables are jointly independent of the third variable. The model function for (AD, M) is:

4 $$log\left( {\mu_{ijk} } \right) = \lambda + \lambda_{i}^{A} + \lambda_{j}^{D} + \lambda_{k}^{M} + \lambda_{ij}^{AD}$$

##### Conditional independence model

This model consists of three possible models with three random variables (AD, AM), (AD, DM) and (AM, DM). Considering the model function for (AD, AM):

5 $$log\left( {\mu_{ijk} } \right) = \lambda + \lambda_{i}^{A} + \lambda_{j}^{D} + \lambda_{k}^{M} + \lambda_{ik}^{AM} + \lambda_{ij}^{AD}$$

Since it contains an AM term, it permits an association between A and M at each category for D. It also permits an AD association at each category of M. It does not contain a DM term, so this log-linear model specifies independence between D and M, at each category for A, that is, conditional independence.

All the log-linear models considered so far are of a special kind, in that whenever there is a higher-order effect in the model, they also include lower-order effects in the model. Such models are called hierarchical log-linear models and are parsimoniously symbolized by a set of top-level terms that uniquely define them (for all variables). The hierarchical log-linear models for three-way tables considered in this study are given in Table 1, along with their notation.

**Table 1:** Hierarchical three-way log-linear models

Model	Model description	log(μ_*ijk*_) =
(A, D, M)	Mutual independence of A, D,M	$\lambda +{\lambda }_{i}^{A}+{\lambda }_{j}^{D}+{\lambda }_{k}^{M}$
Joint independence of		
(D, AM)	D from A and M	$\lambda +{\lambda }_{i}^{A}+{\lambda }_{j}^{D}+{\lambda }_{k}^{M}+{\lambda }_{ik}^{AM}$
(A, DM)	A from D and M	$\lambda +{\lambda }_{i}^{A}+{\lambda }_{j}^{D}+{\lambda }_{k}^{M}+{\lambda }_{jk}^{DM}$
(M, AD)	M from A and D	$\lambda +{\lambda }_{i}^{A}+{\lambda }_{j}^{D}+{\lambda }_{k}^{M}+{\lambda }_{ij}^{AD}$
Conditional independence of		
(AM, DM)	A and D, given M	$\lambda +{\lambda }_{i}^{A}+{\lambda }_{j}^{D}+{\lambda }_{k}^{M}+{\lambda }_{ik}^{AM}+{\lambda }_{jk}^{DM}$
(AD, AM)	D and M, given A	$\lambda +{\lambda }_{i}^{A}+{\lambda }_{j}^{D}+{\lambda }_{k}^{M}+{\lambda }_{ik}^{AM}+{\lambda }_{ij}^{AD}$
(AD, DM)	A and M, given D	$\lambda +{\lambda }_{i}^{A}+{\lambda }_{j}^{D}+{\lambda }_{k}^{M}+{\lambda }_{jk}^{DM}+{\lambda }_{ij}^{AD}$
(AD,AM,DM)	Homogeneous association	$\lambda +{\lambda }_{i}^{A}+{\lambda }_{j}^{D}+{\lambda }_{k}^{M}+{\lambda }_{ik}^{AM}+{\lambda }_{jk}^{DM}$
ADM	Saturated	$\lambda +{\lambda }_{i}^{A}+{\lambda }_{j}^{D}+{\lambda }_{k}^{M}+{\lambda }_{ik}^{AM}+{\lambda }_{ij}^{AD}+{\lambda }_{jk}^{DM}+{\lambda }_{ijk}^{ADM}$

#### Model selection

The log-linear model selection procedure consists of a sequential search among hierarchically nested models. Initiate by testing a saturated model and remove individual terms by conditionally testing their significance. The test statistic increases significantly as the process reaches its end and determines the model to eliminate the following term. At each level of interaction, such as a K-factor interaction, the order in which the interaction terms are removed from the model is in order of importance, with less important terms removed first. The algorithm used in this case is a step-by-step backward elimination method. Alternatively, the forward elimination algorithm assumes a fully independent model and continues adding terms as long as they are conditionally tested and significantly improve the fit.

#### Maximum likelihood estimation for log-linear models

For an $I_{1} \times I_{2} \times ... \times I_{s}$ table cross-classifying variables $X_{1} ,X_{2} ,...X_{s}$ the kernel of the likelihood is:

6 $$\ell \left( \lambda \right) = \,\sum\nolimits_{{i_{1} , \ldots ,i_{s} }}^{s} {n_{{i_{1} , \ldots ,i_{s} }} log\left( {\mu_{{i_{1} , \ldots ,i_{s} }} } \right) - e^{{log\left( {\mu_{{i_{1} , \ldots ,i_{s} }} } \right)}} }$$

where: ${\mu }_{{i}_{1},\dots ,{i}_{s}}$ represents the expected frequencies under the assumed model. $\lambda$ is the vector of all parameters associated with the model.

This equation is maximized with respect to every parameter in $\lambda$, and the set of associated likelihood equations is derived.

For the three-way hierarchical log-linear model (*AM, DM*), for example, Eq. (6) becomes:

7 $$\ell \left( \lambda \right) = \,\sum\nolimits_{{i_{1} ,...,i_{s} }} {n_{{i_{1} ,...,i_{s} }} \left( {\lambda + \lambda_{i}^{A} + \lambda_{i}^{D} + \lambda_{i}^{M} + \lambda_{ik}^{AM} + \lambda_{jk}^{DM} - e^{{\left( {\lambda + \lambda_{i}^{A} + \lambda_{i}^{D} + \lambda_{i}^{M} + \lambda_{ik}^{AM} + \lambda_{jk}^{DM} } \right)}} } \right)}$$

Then solving; $\frac{\partial {\ell}\left(\uplambda \right)}{\partial {\uplambda }_{\text{i}}^{\text{A}}}=0$ gives; ${\widehat{\mu }}_{i++}={n}_{i++}, i=1,\dots ,I$

which are the likelihood equations corresponding to the *A* main effect parameter. The remaining sets of likelihood equations are; ${\widehat{\mu }}_{i++}={n}_{+j+}, j=1,\dots ,J$ and ${\widehat{\mu }}_{++}\text{k}={n}_{++\text{k}}, k=1,\dots ,K$

for the *D* and *M* main effects, respectively. Analogously, with respect to the *AM* interaction parameters,; $\frac{\partial {\ell}\left(\lambda \right)}{\partial {\lambda }_{i}^{A}}=0$ gives;; ${\widehat{\mu }}_{i+k}={n}_{i+k}, i=1,\dots ,I k=1,\dots ,K$ and ${\widehat{\upmu }}_{+\text{jk}}={\text{n}}_{+\text{jk}}, \text{j}=1,\dots ,\text{J k}=1,\dots ,\text{K}$ for the *DM* interaction parameters.

The ML estimates of the λ parameters are:

8 $$\hat{\lambda } = - \frac{1}{{I_{s} }}\sum logn_{s + + } + \frac{1}{{J_{s} }}\sum logn_{ + + s} + \frac{1}{{K_{s} }}\sum logn_{ + s + } - logn$$

9 $$\hat{\lambda }_{i}^{A} = logn_{i + + } - \frac{1}{{I_{s} }}\sum logn_{s + + }$$

10 $$\hat{\lambda }_{j}^{D} = logn_{ + j + } - \frac{1}{{J_{s} }}\sum logn_{ + s + }$$

11 $$\hat{\lambda }_{k}^{M} = logn_{ + + k} - \frac{1}{{K_{s} }}\sum logn_{ + + s}$$

#### Test of conditional independence

In the *I* × *J* × *K* contingency table with classification variables *A*, *D*, and *M*, if the model of homogeneous association (*AD, AM, DM*) fits the data well, it is possible to test for conditional independence between any two of them, given the third. This test will be *conditional* on homogeneous association. For example, the test of

$H_{o}^{{}} :A,D$ are independent, conditional on *M* vs. $H_{1}^{{}} :$ not $H_{o}^{{}}$ can be expressed as:

$H_{o}^{{}} :$ model (*AM, DM*) vs. $H_{1}^{{}}$ model (*AD, AM, DM*)*,*

since we already know that the underlying association is homogeneous. The *H*_0_ and *H*_1_ models are nested; thus, the associated test can be based on the difference $G_{{\text{(AM,DM) }}}^{2} - G_{{\text{(AD, AM, DM)}}}^{2}$ which, under *H*_0_ and given that model (*AD, AM, DM*) holds, is asymptotically distributed as $\chi_{(I - 1)(J - 1)}^{2}$ since *df*(*AM, DM*)* − df*(*AD, AM, DM*) = (*I − *1)(*J − *1).

#### Model fit

The deviance statistic ${G}^{2}$ allows one to evaluate the fit of a log-linear model by comparing the fitted value to a cell to the observed counts. The chi-square $({\chi }^{2})$ degrees of freedom correspond to the number of cells minus the number of model parameters. The deviance is a likelihood ratio statistic to test that all parameters present in the saturated model but not in the working model are equal to zero. These are defined as:

12 $$\chi^{2} = \,\sum\nolimits_{{i_{1} , \ldots ,i_{s} }} {\frac{{\left( {n_{{i, \ldots ,i_{s} }} - \mu_{{i, \ldots ,i_{s} }} } \right)^{2} }}{{\mu_{{i, \ldots ,i_{s} }} }}}$$

13 $$G^{2} = 2\,\sum\nolimits_{{i_{1} , \ldots ,i_{s} }} {\frac{{\left( {n_{{i, \ldots ,i_{s} }} - \mu_{{i, \ldots ,i_{s} }} } \right)^{2} }}{{\mu_{{i, \ldots ,i_{s} }} }}}$$

The residual degrees of freedom $df={d}_{s}-{d}_{r}$ of the hierarchical log-linear models for three-way tables. In this case ${d}_{s}=IJK-1$ and ${d}_{r}$ calculated by adding the number of “free” parameters for the terms in model from Table 2. Given that model2 holds, the adequacy of model1 is tested by ${G}_{model1}^{2}-{G}_{model2}^{2}$ and compared with the ${\chi }_{df\left(model1\right)-df(model2)}^{2}$.

**Table 2:** Hierarchical three-way log-linear models and their residual df

Model	Formula	df
(A, D, M)	(3)	IJK-I-J-K + 2
(D, AM)		(J-1)(IK-1)
(A, DM)		(I-1)(JK-1)
(M, AD)	(4)	(K-1)(IJ-1)
(AM,DM)		K(I-1)(J-1)
(AD, AM)	(5)	I(J-1)(K-1)
(AD, DM)		J(I-1)(K-1)
(AD, AM, DM)	(2)	(I-1)(J-1)(K-1)
(ADM)	(1)	0

#### Evaluating log linear models

Three techniques were employed for assessing log linear models: comparing fitted values, utilizing likelihood ratio statistics, and analyzing cell residuals. Fitted values within log-linear models were contrasted with those of the saturated model. The closer the fitted values to the saturated model, the better the model fit.

*Comparing Fitted Values:* Fitted values within log-linear models were contrasted with those of the saturated model.

*Likelihood Ratio Statistics:* G^2^ statistics were calculated for nested models.

*Residual Analysis:* Cell residuals were analyzed to evaluate model fit.

#### Statistical tests of model fit

*The deviance statistic* ${G}^{2}:$ This assesses goodness-of-fit by comparing observed and expected frequencies. It allows one to evaluate the fit of a log-linear model by comparing the fitted value to a cell to the observed counts. The deviance is a likelihood ratio statistic to test that all parameters present in the saturated model but not in the working model are equal to zero.

*The chi-square* $({\chi }^{2})$: This compares ${G}^{2}$ values between nested models. The chi-square $({\chi }^{2})$ degrees of freedom correspond to the number of cells minus the number of model parameters.

These are defined as:

14 $$\chi^{2} = \,\sum\nolimits_{{i_{1} , \ldots ,i_{s} }} {\frac{{\left( {n_{{i, \ldots ,i_{s} }} - \mu_{{i, \ldots ,i_{s} }} } \right)^{2} }}{{\mu_{{i, \ldots ,i_{s} }} }}}$$

15 $$G^{2} = 2\,\sum\nolimits_{{i_{1} , \ldots ,i_{s} }} {\frac{{\left( {n_{{i, \ldots ,i_{s} }} - \mu_{{i, \ldots ,i_{s} }} } \right)^{2} }}{{\mu_{{i, \ldots ,i_{s} }} }}}$$

*Dissimilarity index Δ:* This measures practical significance of lack of fit*.* It is clear that when n is large, we tend to reject even "good" models. For this purpose, a dissimilarity index is used that assesses the practical significance of the hypothesized lack of model fit in the context of a log-linear model and a large sample size n.

16 $$\Delta = \frac{1}{2n}\,\sum\nolimits_{i = 1}^{I} {\sum\nolimits_{j = 1}^{J} {\sum\nolimits_{k = 1}^{K} {\left| {n_{ijk} - \mu_{ijk} } \right|} } } \, = \,\frac{1}{2}\sum\nolimits_{i = 1}^{I} {\sum\nolimits_{j = 1}^{J} {\sum\nolimits_{k = 1}^{K} {\left| {p_{ijk} - \pi_{jk} } \right|} } }$$

The dissimilarity index Δ lies in the interval [0,1] and represents the percentage of observations that need to be moved to another cell to achieve a perfect fit. Therefore, a smaller value of Δ indicates a better fit.

## Results

### Pattern of malaria, fever, and child anaemia

**Fig. 1:**
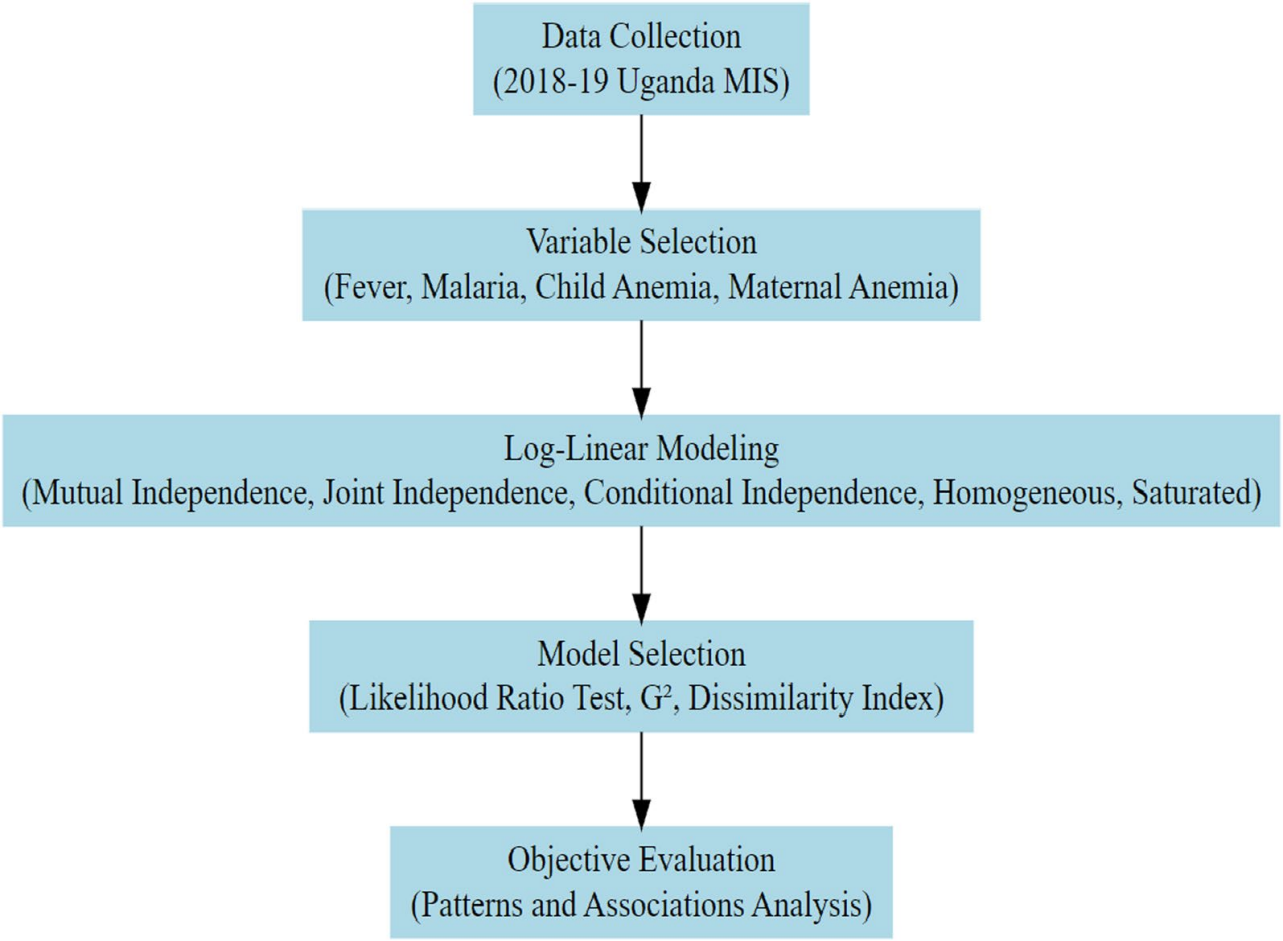
Flowchart summarizing the methodological approach

This section explores the patterns of malaria, fever, and anaemia combinations through summary statistics and visualizations. By employing two tools; a bar chart (Fig. 1A) and a heat map (Fig. 1B), the study aims to provide a clearer and more intuitive understanding of the distribution and relationships between these conditions. These visualizations complement the tabular data (Table 1), highlighting key trends and making the findings more accessible for interpretation.

### Descriptive statistics

Results in Table 1 reveal that out of the 7,124 children in the study, approximately 16% had malaria only, 29% had anaemia only, and 19% reported fever only within the past two weeks. Notably, 13% of children had both malaria and anaemia, while 12% exhibited all three conditions simultaneously. These patterns indicate a substantial overlap between malaria and anaemia, suggesting a syndemic interaction in the study population.

When stratified by maternal anaemia status (Table 1), the most common combination among children of anaemic mothers, was anaemia but no malaria or fever among children (37% of the children). Notably, a significant percentage (16.52%) of children had all three conditions. Therefore, children of anaemic mothers are more likely to exhibit complex health conditions involving multiple illnesses (e.g., Malaria and Anaemia, Malaria, Fever, and Anaemia). This highlights the intergenerational impact of maternal anaemia. This table highlights the significant differences in childhood health outcomes based on maternal anaemia status and hence underscores the need for targeted interventions addressing maternal anaemia to reduce the associated health burden in children.

#### Visualizations

**Fig. 2:**
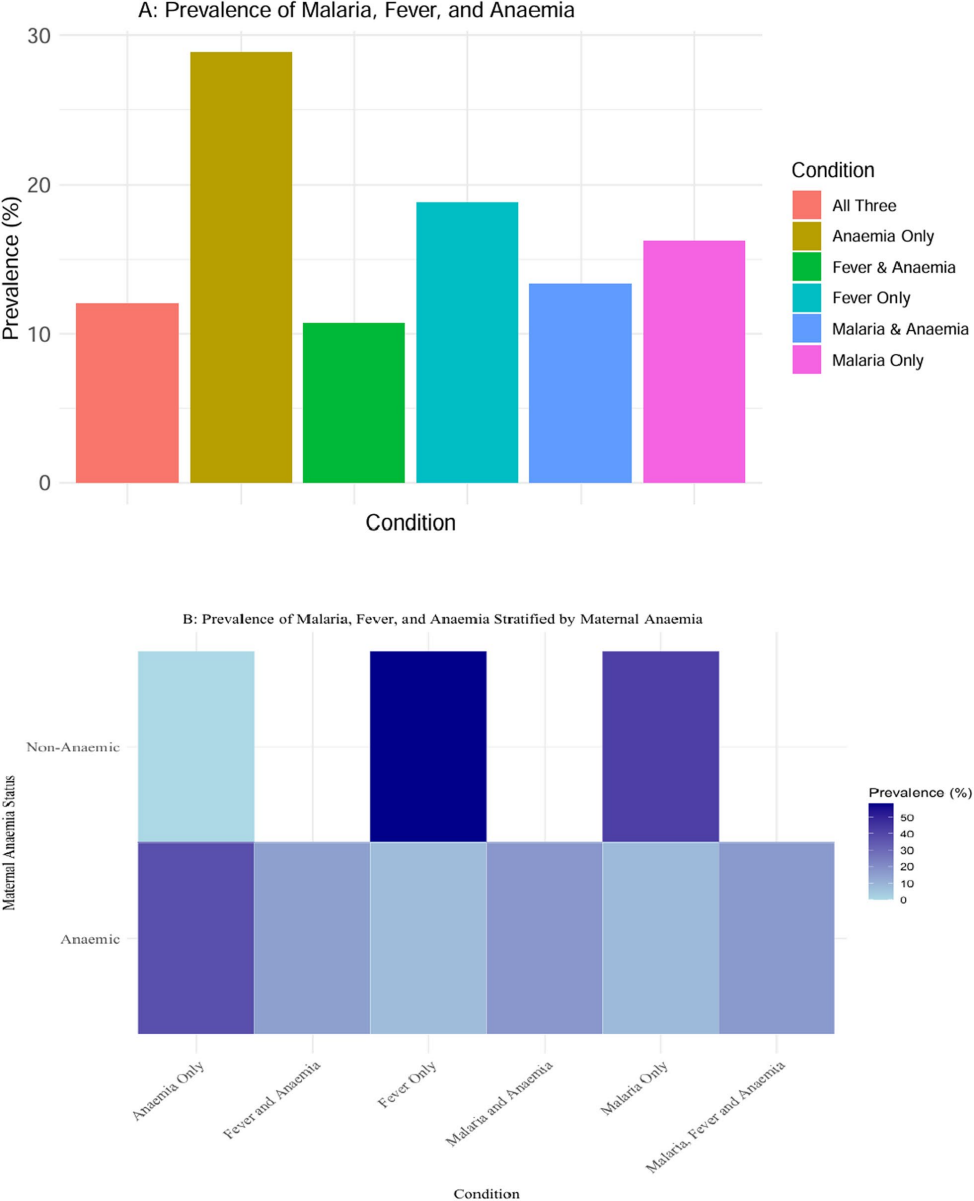
**A** Prevalence of malaria, fever, and anaemia. **B** Prevalence of malaria, fever, and anaemia stratified by maternal anaemia

The bar chart (Fig 2A) illustrates the prevalence of six different conditions, including single conditions (e.g., "Malaria Only," "Fever Only," and "Anaemia Only") and combinations of conditions (e.g., "Malaria & Anaemia" and "All Three") within the study population. The highest prevalence is observed for "Anaemia Only" (28.83%), followed by "Fever Only" (18.84%) and "Malaria Only" (16.24%). The combined conditions have relatively lower prevalence, with "Malaria & Anaemia" (13.36%) and "All Three" (12.01%) being more common than "Fever & Anaemia" (10.72%). These patterns still indicate a substantial overlap between Fever, malaria and anaemia, suggesting a syndemic interaction in the study population.

The heat map (Fig 2B) complements the bar chart by stratifying the prevalence data by maternal anaemia status (Non-Anaemic vs. Anaemic). It shows that combinations of conditions ("Malaria & Anaemia," "Fever & Anaemia," and "Malaria, Fever, and Anaemia") are more prevalent among anaemic individuals compared to non-anaemic individuals, reflecting the heightened risk and vulnerability in this group.

Together, these visualizations provide an integrated view of the data. The bar chart emphasizes the overall distribution of conditions, while the heat map reveals disparities in prevalence based on maternal anaemia status. The combination of these visualizations highlights the critical importance of addressing anaemia as a key driver of health outcomes, particularly when it overlaps with malaria and fever.

### Comparison of log-linear models between Child Anaemia (A), Malaria (D) and Maternal Anaemia (M)

Three techniques are employed for assessing log linear models: comparing fitted values, utilizing likelihood ratio statistics, and analysing cell residuals. Fitted values within log-linear models are contrasted with those of the saturated model. The closer the fitted values are to the saturated model, the better the model fit. Tables 3, 4 shows the fitted values for five log-linear models; mutual independence (A, D, M), joint independence (DM, A), conditional independence (DM, AD), homogeneous (AD, AM, DM) and saturated (ADM).

**Table 3:** Prevalence of malaria, fever, and anaemia combinations

Combination	Percentage
Anaemia only	28.83
Fever only	18.84
Malaria only	16.24
Malaria and anaemia	13.36
Malaria, fever, and anaemia	12.01
Fever and anaemia	10.72

**Table 4:** Prevalence of malaria, fever, and anaemia combinations stratified by maternal anaemia

Maternal anaemia	Combination	Percentage
Negative	Fever only	57.99
Negative	Malaria only	42.01
Positive	Anaemia only	37.42
Positive	Malaria and anaemia	16.94
Positive	Malaria, fever, and anaemia	16.52
Positive	Fever and anaemia	14.62
Positive	Malaria only	7.73
Positive	Fever only	6.78

### Fitted values for log linear models

Tables 3, 4 presents a comparison of the fitted values from various log-linear models against the saturated model (ADM). The findings reveal that the homogeneous model (AD, AM, DM) exhibits the closest fitted values to the saturated model, suggesting it's the most suitable model compared to others. Conversely, the other models demonstrate poor fit, evidenced by significant disparities between their fitted values and those of the saturated model.

### Likelihood ratio and goodness of fit (${\mathbf{G}}^{2}$) test and dissimilarity index, $\widetilde{\Delta }$

A better model fit is indicated by a likelihood ratio (${G}^{2}$) statistic value that is lower. Similarly, a smaller value of $\widetilde{\Delta }$ indicates a better fit. The likelihood ratio goodness of fit (${G}^{2}$) statistics results for each model and corresponding values of $\widetilde{\Delta }$ are presented in Table 5.

**Table 5:** Fitted values for log linear models

Maternal anaemia (M)	Child malaria (D)	Child anaemia (A)	Log linear model				
			Mutual independence (A, D, M)	Joint independence (AD, M)	Conditional independence (AM, DM)	Homogenous (AD, AM, DM)	Saturated (ADM)
Anaemic	Positive	Anaemic	339.38	440.97	879.00	879	879
		Not anaemic	634.19	824.03	264.52	386	386
	Negative	Anaemic	841.31	739.72	1367.00	1367	1367
		Not anaemic	1572.12	1382.3	876.48	755	755
Not anaemic	Positive	Anaemic	306.22	204.63	0.00	0.00	0
		Not anaemic	572.21	382.37	708.48	587	587
	Negative	Anaemic	759.09	860.68	0.00	0.00	0
		Not anaemic	1418.48	1608.3	2347.52	2469	2469

Table 5 shows that the most fitting model is the homogeneous model (AD, AM, DM). This is because; other than the Saturated model (ADM), the homogeneous model has the smallest ${G}^{2}$ statistic (${G}^{2}$= 1.74e-06) and largest p-value (= 0.999). In addition, it has the smallest dissimilarity index $\widetilde{\Delta } <0.001$; stating that less than 0.1% of the observations must be moved to achieve a perfect fit.

### Log linear cell residual

The larger residual indicates the data is poorly fit. The smaller positive and negative residuals indicate strength between a specific relationship, while larger positive and negative residuals indicate a feeble association. Tables 3, 4 shows the log-linear cell residual for the homogeneous model (AD, AM, DM).

Table 6 shows that the standardized residual values are very small. There is no extreme difference between the fitted and observed values. Both the positive and negative residuals' values are small, indicating a decent model fit. At long last, the homogeneous model of Child Anaemia, Malaria, and Maternal Anaemia is found to be the most parsimonious model contrasted with other log-linear models.

**Table 6:** Likelihood ratio goodness of fit statistics and dissimilarity index

Model	${G}^{2}$	Degree of freedom	P-value	$\widetilde{\Delta }$
A, D, M	4276.03	4	0.000	0.331
AD, M	4012.72	3	0.000	0.331
AM, DM	94.78	2	0.000	0.038
AD, AM, DM	1.74e-06	1	0.999	< 0.001
ADM	0	0	1.000	< 0.001

### Comparison of log linear models between Child Anaemia (A), Fever (F) and Maternal Anaemia (M)

15 $$log\left( {\mu_{ijk} } \right) = \lambda + \lambda_{i}^{A} + \lambda_{j}^{F} + \lambda_{k}^{M} + \lambda_{ik}^{AM} + \lambda_{jk}^{FM} + \lambda_{ij}^{AF} + \lambda_{ijk}^{AFM}$$

### Fitted values

Table 7 presents a comparison of the fitted values from various log-linear models against the saturated model (AFM). The findings reveal that the homogeneous model (AF, AM, FM) exhibits the closest fitted values to the saturated model, suggesting it's the most suitable model compared to others. Conversely, the other models demonstrate poor fit, evidenced by significant disparities between their fitted values and those of the saturated model.

**Table 7:** Log linear cell residuals for the homogeneous model

Maternal anaemia (M)	Child malaria (D)	Child anaemia (A)	Observed count	Log linear model (AD, AM, DM)	
				Fitted count	Standardized residual
Anaemic	Positive	Anaemic	879	879	0.00000052
		Not anaemic	386	386	−0.00000030
	Negative	Anaemic	1367	1367	0.00000032
		Not anaemic	755	755	0.00000028
Not anaemic	Positive	Anaemic	0	0.00	−0.00000734
		Not anaemic	587	587	0.00000021
	Negative	Anaemic	0	0.00	−0.00002454
		Not anaemic	2469	2469	−0.00000127

### Likelihood ratio and goodness of fit (${\mathbf{G}}^{2}$) test and dissimilarity index, $\widetilde{\Delta }$

Table 8 shows that the most fitting model is the homogeneous model (AF, AM, FM). This is on the grounds that; other than the Saturated model (AFM), the homogeneous model has the smallest ${G}^{2}$ statistic (${G}^{2}$= 2.13e-06) and largest p = 0.999. In addition, it has the smallest dissimilarity index $\widetilde{\Delta } <0.001$; stating that less than 0.1% of the observations must be moved to achieve a perfect fit.

**Table 8:** Fitted values for log linear models

Maternal anaemia (M)	Child fever (D)	Child anaemia (A)	Log linear model				
			(A, F, M)	(AF, M)	(AM, FM)	(AF, AM, FM)	(AFM)
Anaemic	Yes	Anaemic	293.95	365.80	818.00	818.00	818
		Not anaemic	653.48	813.20	295.11	361.00	361
	No	Anaemic	645.51	573.67	1069.00	1069.00	1069
		Not anaemic	1435.05	1275.33	845.89	780.00	780
Not anaemic	Yes	Anaemic	296.47	224.63	0.00	0.00	0
		Not anaemic	659.09	499.37	789.89	724.00	724
	No	Anaemic	651.06	722.91	0.00	0.00	0
		Not anaemic	1447.37	1607.09	2264.11	2330.00	2330

### Log linear cell residual

Table 9 shows that the standardized residual values are very small. There is no extreme difference between the fitted and observed values. Both the positive and negative residuals' values are small, indicating a decent model fit. At long last, the homogeneous model of Child Anaemia, Fever and Maternal Anaemia is found to be the most parsimonious model contrasted with other log linear models.

**Table 9:** Likelihood ratio goodness of fit statistics and dissimilarity index

Model	${G}^{2}$	Degree of freedom	P-value	$\widetilde{\Delta }$
A, F, M	3728.02	4	0.000	0.312
AF, M	3562.79	3	0.000	0.312
AM, FM	26.55	2	< 0.001	0.022
AF, AM, FM	2.13e-06	1	0.999	< 0.001
AFM	0	0	1.000	< 0.001

### Test of effect sizes on the most parsimonious model

#### Parameter estimates for child anaemia (A), child malaria (D), maternal malaria (M)

Table 10 shows the parameter estimates for the most parsimonious homogenous model (AD, AM, DM). The parameters estimates were obtained from fitting the homogeneous log linear model as follows:

16 $$\begin{gathered} \log \left( {\mu_{ijk} } \right) = \lambda + \lambda_{i}^{A} + \lambda_{j}^{D} + \lambda_{k}^{M} + \lambda_{ik}^{AM} + \lambda_{jk}^{DM} + \lambda_{ij}^{AD} \hfill \\ \quad \quad \quad \;\;\; = 7.812 - 1.185A - 1.437D - 30.510M - 0.766AD + 31.1AM - 0.229DM \hfill \\ \end{gathered}$$

**Table 10:** Log linear cell residuals for the homogeneous model

Maternal anaemia (M)	Child fever (F)	Child anaemia (A)	Observed count	Log linear model (AF, AM, FM)	
				Fitted count	Standardized residual
Anaemic	Yes	Anaemic	818	818.00	0.00000084
		Not Anaemic	361	361.00	−0.00000028
	No	Anaemic	1069	1069.00	0.00000057
		Not Anaemic	780	780.00	0.00000026
Not anaemic	Yes	Anaemic	0	0.00	−0.00000907
		Not Anaemic	724	724.00	−0.00000043
	No	Anaemic	0	0.00	−0.00001765
		Not Anaemic	2330	2330.00	−0.00000037

For parameters interaction ${\lambda }_{ij}^{AD}$ Anaemia and Malaria status of the child, the estimated value is 0.766 with a p < 0.01), and the odds ratio (${e}^{0.766}$) equals 2.15.

The value 2.15 indicates that for a child who tested Malaria positive, the estimated odds of Anaemia infection are 2.15 times the estimated odds of those who tested Malaria negative; controlling for Maternal Anaemia status.

#### Parameter estimates for child anaemia (A), child fever (F), maternal malaria (M)

Table 11 shows the parameter estimates for the most parsimonious homogenous model (AF, AM, FM). The parameters estimates were obtained from fitting the homogeneous log linear model as follows:

17 $$\begin{gathered} \log \left( {\mu_{ijk} } \right) = \lambda + \lambda_{i}^{A} + \lambda_{j}^{F} + \lambda_{k}^{M} + \lambda_{ik}^{AM} + \lambda_{jk}^{FM} + \lambda_{ij}^{AF} \hfill \\ \quad \quad \quad \;\;\, = 7.754 - 1.094A - 1.169F - 30.72M + o.398AF + 31.04AM + 0.503FM \hfill \\ \end{gathered}$$

**Table 11:** Parameter estimates for fitting the homogeneous model (AD, AM, DM)

Parameter	Estimate	Std. Error	Z value	P-value
(Intercept)	7.812	0.020	388.1	< 2e-16
A = anaemic	−1.185	0.042	−28.49	< 2e-16
D = positive	−1.437	0.046	−31.28	< 2e-16
M = anaemic	−30.51	45190	−0.001	0.999
[A = anaemic]*[D = positive]	0.766	0.078	9.865	< 2e-16
[A = anaemic]*[M = anaemic]	31.10	45190	0.001	0.999
[D = positive]*[M = anaemic]	−0.229	0.076	3.015	0.003

For parameters interaction ${\lambda }_{ij}^{AF}$ Anaemia and Fever status of the child, the estimated value is 0.398 with a p-value = 0.000 (< 0.01), and the odds ratio (${e}^{0.398}$) equals 1.49.

The value 1.49 indicates that for a child who had Fever in the last two weeks, the estimated odds of Anaemia infection are 1.49 times the estimated odds of those who had no Fever; controlling for Maternal Anaemia status (Table 12).

**Table 12:** Parameter estimates for fitting the homogeneous model (AF, AM, FM)

Parameter	Estimate	Std. Error	Z value	P-value
(Intercept)	7.754	0.021	374.26	< 2e-16
A = anaemic	−1.094	0.041	−26.45	< 2e-16
F = yes	−1.169	0.043	−27.47	< 2e-16
M = anaemic	−30.72	47930	−0.001	0.999
[A = anaemic]*[F = yes]	0.398	0.077	5.203	0.000
[A = anaemic]*[M = anaemic]	31.04	47930	0.001	0.999
[F = yes]*[M = anaemic]	0.503	0.079	6.380	0.000

## Discussion and conclusion

This study explored the associations between malaria, fever, and anaemia in children while accounting for maternal anaemia, using log-linear modelling to uncover patterns and interactions among these conditions. The findings not only align with previous research but also address critical gaps in understanding how these conditions interact in resource-limited settings such as Uganda. Below, the results are situated within the broader literature, exploring disparities, and highlighting their contribution to existing knowledge.

The descriptive statistics and visualizations in the results section of this study provide critical insights into the prevalence and interactions of malaria, fever, and anaemia among children in Uganda, with maternal anaemia as a key contextual factor. The bar chart underscores that single conditions, particularly anaemia, are the most prevalent health issues, with "Anaemia Only" observed in 28.83% of cases. Conditions such as "Malaria Only" and "Fever Only" also exhibit substantial prevalence, highlighting their individual contributions to the burden of disease. Conversely, combinations of conditions, including "Malaria & Anaemia" and "All Three," although less prevalent, demonstrate the complex interdependencies among these health outcomes.

The heat map offers further detail by stratifying prevalence data by maternal anaemia status. The visualizations reveal that maternal anaemia significantly influences the distribution of conditions among children. Non-anaemic mothers tend to have children with higher prevalence of "Fever Only" and "Malaria Only," while children of anaemic mothers are more likely to experience "Anaemia Only" and combinations of conditions like "Malaria & Anaemia." These patterns suggest that maternal anaemia not only amplifies the risk of childhood anaemia but also alters the co-occurrence dynamics of malaria and fever.

These results are consistent with the syndemic framework, which posits that overlapping health conditions in resource-limited settings interact synergistically to exacerbate health outcomes. The visualizations emphasize the need for integrated approaches to address these interconnected health issues. Furthermore, they align with prior evidence, such as the strong association between malaria and anaemia, while also shedding light on the nuanced role of maternal anaemia in shaping these interactions.

The finding that children with malaria are over twice as likely to have anaemia (odds ratio = 2.15) aligns with extensive evidence from sub-Saharan Africa. For instance, studies conducted in Ghana, Kenya, and Nigeria have consistently shown a strong association between malaria and anaemia in children under five years of age [22–24]. Malaria’s direct contribution to anaemia—through mechanisms such as hemolysis, reduced erythropoiesis, and bone marrow suppression—is well-documented, further supporting the results [25]. Similarly, the observation that children with recent fever are 1.5 times more likely to have anaemia echoes findings from a study among Palestinian refugee children, which linked febrile illnesses to a higher risk of anaemia due to inflammatory responses and nutritional depletion [26].

However, fewer studies have explicitly considered the influence of maternal anaemia on childhood anaemia in the context of malaria and fever. These results demonstrate that maternal anaemia serves as an important contextual factor, indirectly contributing to the child’s risk of anaemia through intergenerational health effects, including shared nutritional deficiencies and poor perinatal health. This complements findings from Ethiopia and Tanzania, which have noted the role of maternal health in shaping child morbidity, though without explicitly examining maternal anaemia in relation to malaria and fever [27, 28].

While the findings of the study align with the general consensus on the malaria-anaemia relationship, there are notable differences in effect sizes and observed prevalence rates compared to other regions. For example, anaemia prevalence (53%) is higher than that reported in some parts of sub-Saharan Africa, such as Senegal (42%) and Rwanda (38%) [29, 30]. These differences may be attributable to variations in malaria endemicity, nutritional profiles, and healthcare access. Uganda’s persistent malaria burden and high prevalence of iron deficiency anaemia likely amplify the interaction between these conditions [2, 31]. Additionally, socioeconomic factors, such as food insecurity and limited access to health services, further exacerbate disparities in child health outcomes.

Differences in study design may also account for observed disparities. For instance, cross-sectional surveys, such as the Demographic and Health Surveys (DHS), provide a snapshot of health conditions, whereas the use of log-linear modelling allowed us to rigorously explore the interactions between conditions while controlling for confounders like maternal anaemia. Simpler statistical methods used in some other studies may overlook these nuanced interactions, which highlights the value of the approach [32].

This study makes several important contributions to the literature. First, log-linear modelling provides a robust statistical framework to analyse the complex relationships between malaria, fever, and anaemia in children. Unlike traditional methods, this approach captures interactions and dependencies among variables, enabling a deeper understanding of how these conditions influence each other.

Second, inclusion of maternal anaemia as a control variable adds a new dimension to the study of childhood anaemia. While previous research has focused primarily on child-specific factors, the findings emphasize the critical role of maternal health in shaping child morbidity. This supports the syndemic framework, which posits that overlapping health conditions in resource-limited settings act synergistically to worsen outcomes [19].

Finally, the study addresses a significant gap in the literature by highlighting the bidirectional nature of the malaria-anaemia relationship. Although many studies have documented that malaria contributes to anaemia, fewer have explored how anaemia, in turn, increases susceptibility to malaria. This reciprocal interaction underscores the need for integrated interventions that address both conditions simultaneously. For example, malaria prevention strategies, such as the distribution of insecticide-treated bed nets, could significantly reduce anaemia prevalence by lowering malaria incidence. Similarly, addressing nutritional deficiencies that contribute to anaemia may indirectly reduce malaria susceptibility by improving immune function [33, 34].

The findings have important implications for public health policy and intervention design. Integrated approaches that simultaneously target malaria, anaemia, and fever are critical for reducing the burden of childhood morbidity in Uganda. For instance, combining malaria prevention efforts with nutritional supplementation and maternal health programmes could yield significant synergistic benefits. Additionally, the identification of maternal anaemia as a contextual factor highlights the need for interventions that address intergenerational health challenges [19].

Future research should build on these findings by exploring longitudinal relationships between these conditions. While the cross-sectional design provides valuable insights, a prospective cohort study could better capture the temporal dynamics and causal pathways underlying these interactions. Furthermore, exploring the role of other co-factors, such as intestinal parasitic infections and socioeconomic determinants, could provide a more comprehensive understanding of childhood anaemia and its drivers.

In conclusion, this study highlights the interconnectedness of malaria, fever, and anaemia in Ugandan children, while underscoring the importance of maternal anaemia as a contextual factor. These findings underscore the importance of targeting maternal health and developing integrated interventions to address this syndemic in resource-limited settings like Uganda.

## Data Availability

No datasets were generated or analysed during the current study.
